# Computational hemodynamic pathophysiology of internal carotid artery blister aneurysms

**DOI:** 10.1186/s12938-024-01306-z

**Published:** 2024-11-21

**Authors:** Tristan Martin, Gilles El Hage, Claude Barbeau, Michel W. Bojanowski

**Affiliations:** Division of Neurosurgery, Centre Hospitalier de L’Université de Montréal, Montréal, QC Canada

**Keywords:** Blister aneurysm, Carotid siphon, Computational fluid dynamics, Sidewall aneurysm, Wall shear stress

## Abstract

**Objective:**

Blister aneurysms of the internal carotid artery (ICA) are rare and are primarily documented in the literature through small series and case reports. The intraoperative observation of a hemorrhage in the artery wall proximal to the aneurysmal bulge led to the hypothesis that some of these aneurysms might develop in a retrograde manner.

**Methods:**

We developed software to reconstruct the ICA with and without Type I and II blister aneurysms using patients’ imagery as input to simulate hemodynamic conditions before and after their formation. Kinematic blood flow data before and after aneurysm formation were obtained using a finite volume solver. We compared the wall shear stress (WSS) distribution of the arterial wall prior to aneurysm formation.

**Results:**

In two out of four cases, WSS was significantly elevated on the dorsal wall of the supraclinoid segment of the ICA at the distal part of the future site of the aneurysm sac, suggesting that the aneurysm sac may ultimately develop in a retrograde fashion. Once the structural changes have been initiated, WSS gradient (WSSG) was significantly elevated at the proximal and distal boundaries of the bulging aneurysmal pouch. Low WSS and high WSSG at the proximal part of the aneurysm sac seem to contribute to the extension of the proximal intramural hematoma observed during blister aneurysm surgery.

**Conclusions:**

By enabling assessment of the impact of elevated WSS and its gradient, our computational pipeline supports the hypothesis that the development of blister aneurysms may occur either in a retrograde or anterograde fashion.

## Background

**Fig. 1:**
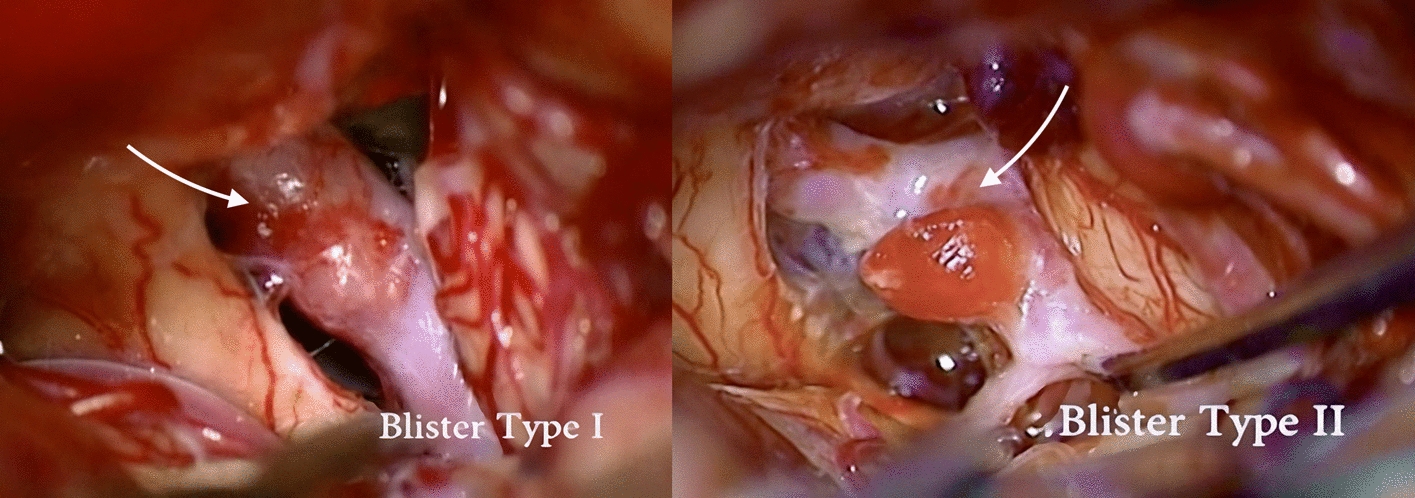
Surgical view of Type I and Type II blister aneurysms of the ICA. The intramural hematoma proximal to the bulging aneurysmal pouch is indicated with a white arrow

Blister aneurysms of the internal carotid artery (ICA) are rare sidewall lesions typically arising at non-branching sites of the dorsal wall of the supraclinoid segment (C6 in Bouthillier’s classification [1] or C2 in Fischer’s classification [2]) of the ICA [3, 4]. Surgical series rarely surpass ten patients [5]. These dissecting aneurysms, characterized by their thin walls, traditionally pose a high risk of morbidity and mortality, regardless of whether they are treated surgically or endovascularly [6]. Yet, the hemodynamic factors driving their pathogenesis are poorly understood [7]. Upon reviewing the surgical video series on blister aneurysms of the ICA, the senior author (M.W.B) observed that in certain cases, the vascular hemorrhage within the wall of the parent artery extended beyond the proximal side of the aneurysmal pouch, as shown in Fig. 1. This led to the suggestion that these aneurysms might develop in a retrograde fashion in some patients. In order to assess this hypothesis and investigate other mechanisms of blister aneurysm progression, we used computational fluid dynamics (CFD) to investigate the distribution of wall shear stress (WSS) contributing to the mechanisms of blister formation and progression.

## Results

**Fig. 2:**
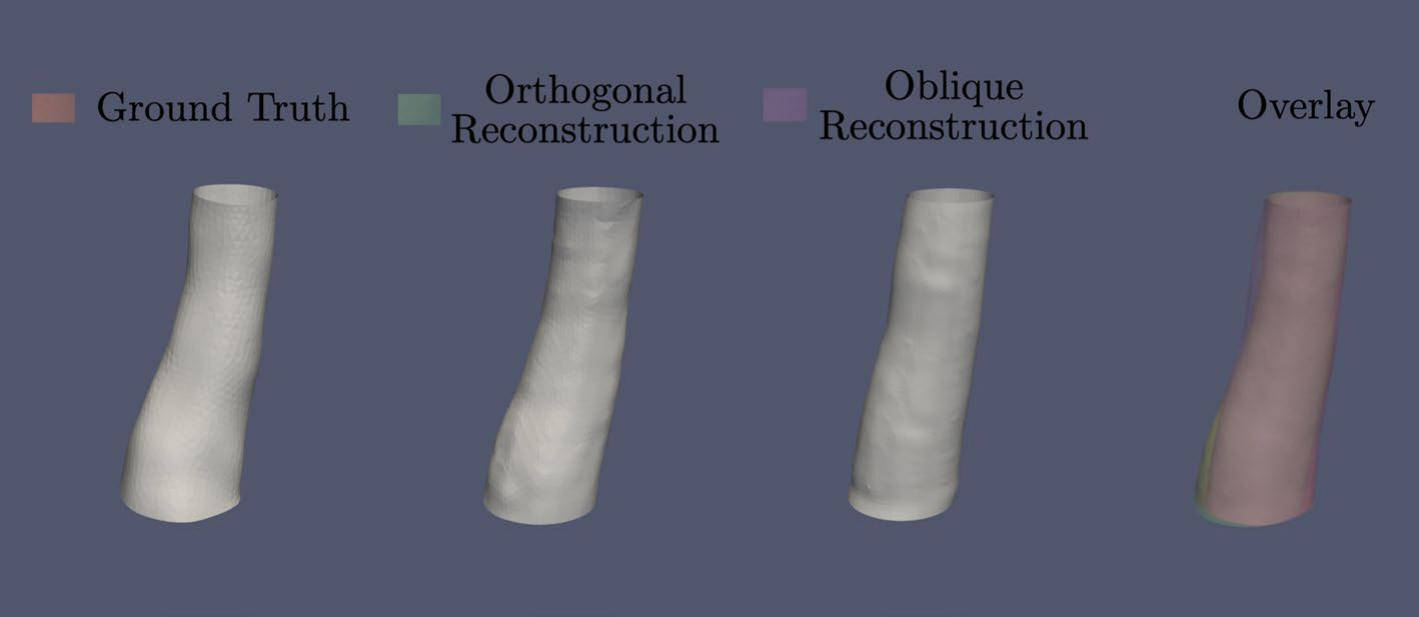
Simulated X-ray angiographic reconstruction and ground truth models of the supraclinoid ICA. Both methodologies using orthogonal and oblique projections as input provide reliable reconstruction of the ICA as seen on the overlay

**Fig. 3:**
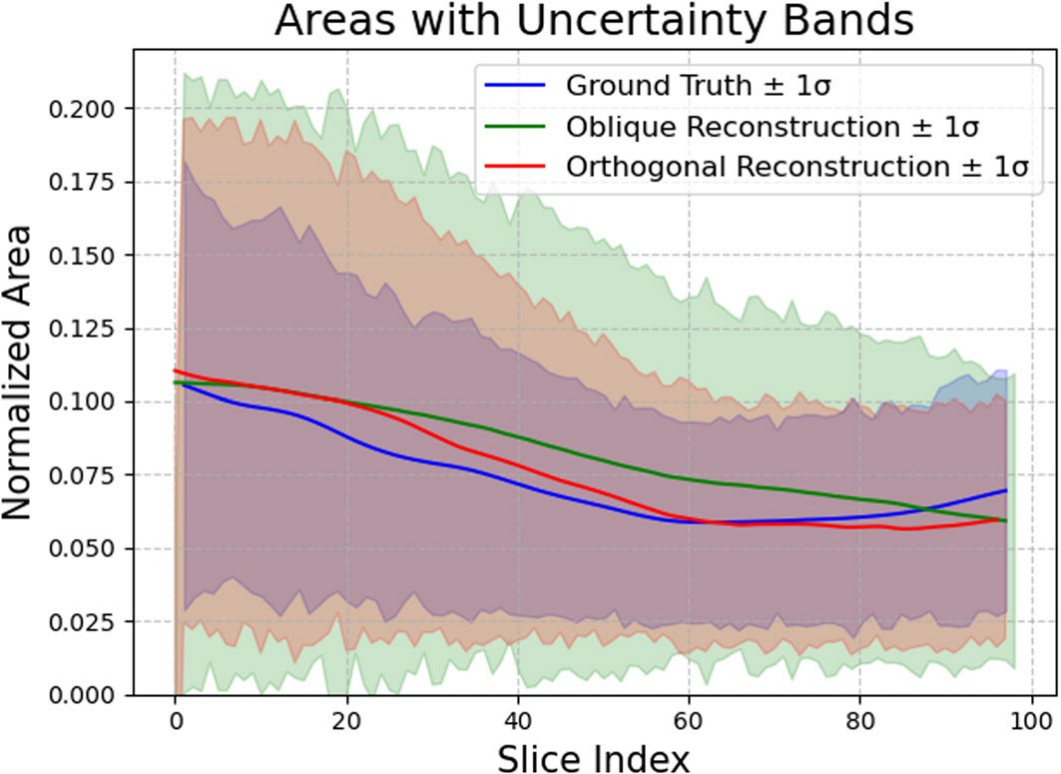
Cross-sectional area measurements along the Z-axis for both oblique and orthogonal reconstructions compared to the ground truth data. Observed cross-sectional area for the oblique and orthogonal reconstructions along the Z-axis fall within the uncertainty interval of the ground truth

Both methods for X-ray angiographic reconstruction of the ICA produced reliable models when compared with the ground truth (Fig. 2). In fact, the measured values for cross-sectional area for both the oblique and orthogonal reconstructions along the Z-axis fall within the uncertainty interval of the ground truth data (Fig. 3).

For all simulations hereby presented, our model demonstrated excellent steady-state convergence with residuals for 3D velocity components and pressure reaching less than ${10}^{-5}$.

Pressure and WSS are presented in kinematic units (SI units normalized by blood density) to enable direct comparisons with simulations conducted by other groups using varying blood densities.

**Fig. 4:**
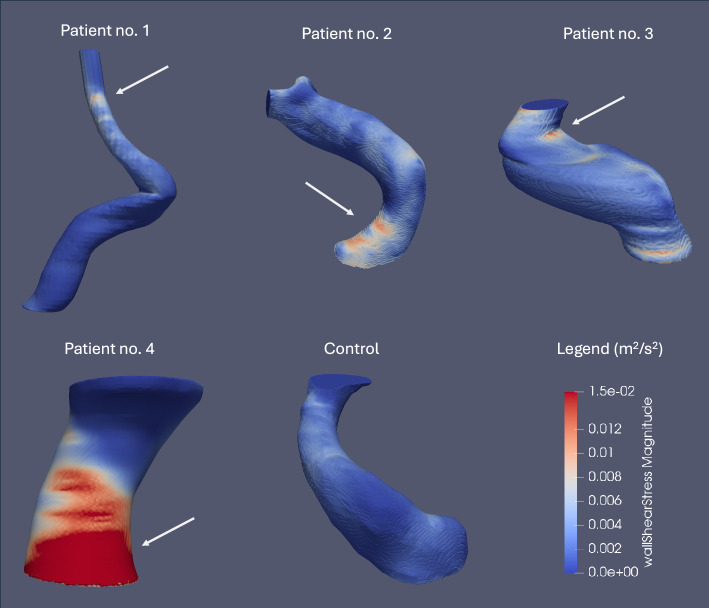
WSS distributions in kinematic units (${\text{m}}^{2}/{\text{s}}^{2}$) in the control patient and in patients with blister aneurysms before aneurysm formation under normotensive conditions (MAP = 90 mmHg) in all patients. In blisters no. 1 and 3, WSS is significantly elevated on the dorsal wall of the supraclinoid segment of the ICA at the distal part of the future site of the aneurysm sac. In the case of the remaining two patients, we observed a significant elevation in WSS at, or proximal to, the site of the future aneurysm sac. The control case showed no regions of significantly elevated WSS. Regions of significantly elevated WSS on the dorsal wall of the C6 segment of the ICA are indicated with a white arrow

In two patients with blister aneurysms, WSS was considerably elevated (> 10 Pa or $\approx$ 0.095 ${m}^{2}/{s}^{2}$) on the dorsal wall of the supraclinoid segment of the ICA at the distal part of the future site of the aneurysmal sac, suggesting a retrograde development of the aneurysm (Fig. 4). In the remaining two patients, WSS was significantly elevated at and proximal to the region where the aneurysmal sac eventually formed (Fig. 4). The control ICA displayed no regions of elevated WSS (Fig. 4).

**Fig. 5:**
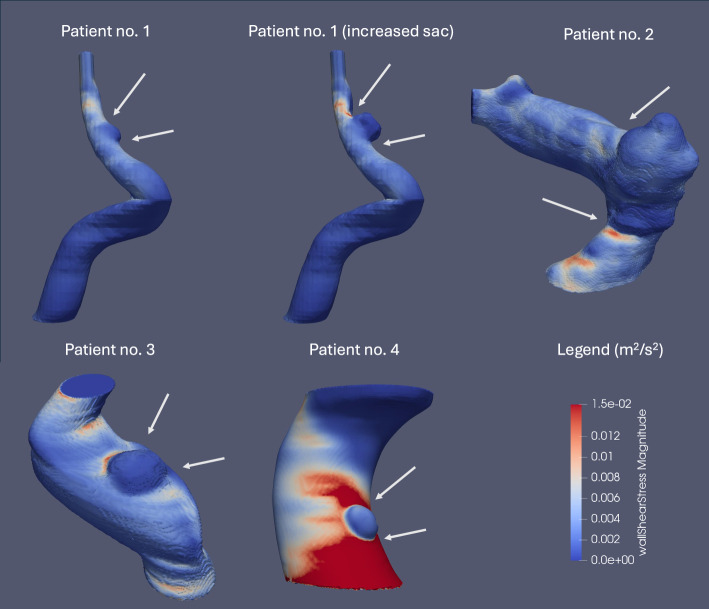
WSS distributions in kinematic units (${m}^{2}/{s}^{2}$) after aneurysm formation under normotensive conditions (MAP = 90 mmHg) in all patients. We observe very low intrasaccular WSS associated with a high WSSG at the boundary of the aneurysmal sac, which may favor extension of the dissection. Regions of elevated WSSG are indicated with a white arrow

On the other hand, WSSG was elevated at both the proximal and distal boundaries of the bulging aneurysmal pouch, which may constitute an important hemodynamic driver for aneurysmal progression (Fig. 5). Moreover, very low intrasaccular WSS associated with a high WSSG at the proximal part of the aneurysm sac may explain the extension of the intramural hematoma of the ICA proximal to the forming blister.

**Fig. 6:**
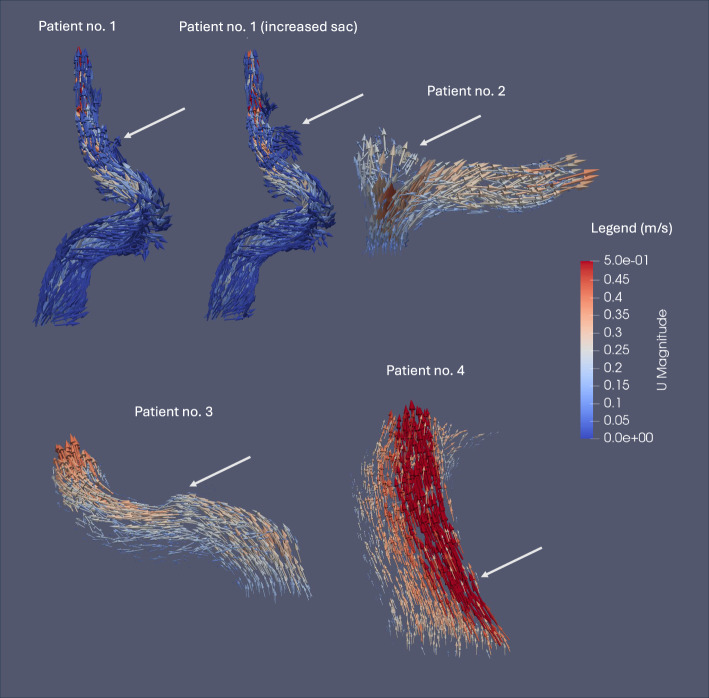
Streamlines ($\text{m}/s$) under normotensive conditions (MAP = 90 mmHg) for the original and enhanced case 1 as well as for cases no. 2, 3, and 4. In patient no. 1, the initial anterograde flow transitions to retrograde recirculation within a larger digitally increased sac. On the other hand, in patient no. 2, strong anterograde flow is observed along the lateral aspect, with part of the flow decelerating and forming a vortex on the medial aspect, despite the presence of a sizable aneurysmal sac. Initial blister aneurysms no. 3 and 4, characterized by a small outpouching, show anterograde flow. The intrasaccular flow patterns are indicated with a white arrow

Characterized by a small outpouching or a berry-like sac, Type I and II blister aneurysms featured in our series all initially exhibited anterograde flow patterns (Fig. 6). On the other hand, our CFD study demonstrated intrasaccular retrograde recirculation after digitally increasing the sac of blister no. 1 (Fig. 6), as is well documented in the literature in certain cerebral sidewall aneurysms [8–12]. For blister no. 2, where anterograde development is suspected from the WSS distribution before aneurysmal pathogenesis, the major intrasaccular flow pattern persisted in the anterograde direction (Fig. 6) even in the presence of a sizable sac.

**Fig. 7:**
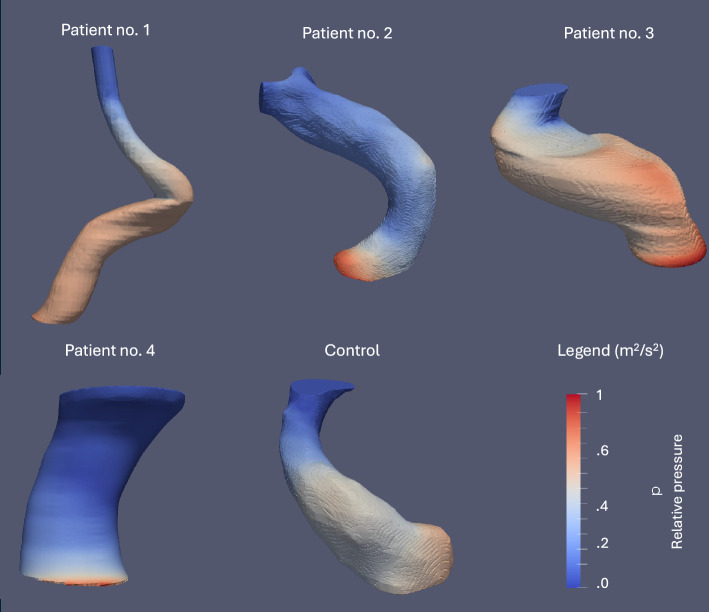
Relative pressure distributions in kinematic units (${m}^{2}/{s}^{2}$) under normotensive conditions (MAP = 90 mmHg) before aneurysm formation in all patients. Regions of high blood pressure on the vessel wall show no association with the site of the future aneurysmal pouch. The pressure field was normalized such that the relative pressure difference between the inlet and the outlet is 1 kinematic unit, with the zero mark and the upper limit corresponding to 11 and 12 absolute kinematic units

Moreover, regions of high blood pressure had no predictive power for the future site of the aneurysm (Fig. 7). The contributions of dynamic pressure, $\frac{1}{2}\uprho {v}^{2},$ and hydrostatic pressure, $\uprho g\Delta h,$ which were less than the order of 5 mmHg and may be subject to variations depending on position, were negligible in comparison to static pressure.

## Discussion

The presence of proximal intramural hematomas observed surgically in the treatment of some blister aneurysms [5, 13, 14] possibly hints at a potential pathophysiological mechanism involving retrograde formation and progression in these lesions. While it is known that intrasaccular retrograde recirculation may occur in sidewall aneurysms [8–12], its influence in the initiation and growth of blister aneurysms of the ICA remains poorly understood.

The formation and progression of intracranial aneurysms are influenced significantly by hemodynamic states characterized by abnormal WSS, which can arise from factors such as arterial morphology and hypertension [15, 16]. Shear stress is known to play a critical role in the pathogenesis of cerebral aneurysms by causing endothelial cell dysfunction, ultimately eliciting an inflammatory response that compromises the integrity of the vessel wall [17]. While this hemodynamic environment is well-documented for saccular aneurysms, its applicability to blister aneurysms requires careful consideration.

Unlike saccular aneurysms, which are characterized by a sac with an endothelial layer, blister aneurysms are localized wall defects covered only by a thin fibrous tissue, akin to dissecting aneurysms, and do not possess the same structural features as true aneurysms [3]. The mechanical stimulus of WSS is sensed by endothelial cells in saccular aneurysms, leading to mechanotransduction and chronic inflammation. However, in blister aneurysms, the absence of endothelial cells and vascular media necessitates the exploration of alternative mechanisms of hemodynamic involvement. It is plausible that the mechanisms underlying blister aneurysm formation may not involve chronic inflammation mediated by endothelial cell sensing of blood flow but could instead relate to the direct physical effects of mechanical stress.

Histopathological studies have demonstrated distinct differences between the biomolecular environments of saccular and blister aneurysms [18]. Although some similarities in inflammatory responses exist, blister aneurysm tissue is characterized by acute inflammation, increased cell apoptosis, and elevated levels of proliferation, as opposed to the chronic inflammatory processes seen in saccular aneurysms [18]. Additionally, preexisting intracranial atherosclerosis has been shown to predispose individuals to the development of blister aneurysms, suggesting a different pathophysiological mechanism than that observed in saccular aneurysms [19].

Overall, while hemodynamic involvement in blister aneurysms is acknowledged, it is essential to delineate the differences in mechanisms from those observed in saccular aneurysms to accurately reflect the current understanding of these distinct entities. Due to their similarities with dissecting aneurysms, the pathogenesis and progression of blister aneurysms may be more directly influenced by elevated WSS and WSSG, rather than by the mechanotransduction and chronic inflammation mechanisms typically associated with saccular aneurysms.

Specifically, while very high WSS (near or above 10 Pa [20] or ${10}^{-2} {m}^{2}/{s}^{2}$) induces supraphysiological endothelial cell turnover and increased tPA production in saccular aneurysms, low stress (< 0.4 Pa or ${4 \times 10}^{-4} {m}^{2}/{s}^{2}$ [21]) exacerbates cell apoptosis, stasis, and low tPA production, consequently favoring thrombogenesis [17, 22]. On the other hand, normal WSS (around 2 Pa or ${2 \times 10}^{-4} {m}^{2}/{s}^{2}$ [20]) is necessary for endothelial cell homeostasis. In fact, CFD and MRI studies report normal ICA WSS to be $1.07 \pm 0.52$ Pa and $0.73 \pm 0.26$ Pa, respectively [23].

Our study suggests that prior to aneurysm formation, hemodynamic WSS was significantly elevated, for two patients out of four, on the dorsal wall of the supraclinoid segment of the ICA at the distal part of the future site of the aneurysm sac. In all cases, regions of supraphysiological WSS on the dorsal wall of the ICA were correlated with the future site of the blister aneurysm. The absence of such regions on the control ICA underscores the critical role that elevated WSS may play in the development of blister aneurysms.

Our study suggests that, in this region, blister aneurysms may ultimately progress retrogradely due to the morphology of the supraclinoid ICA. However, in the case of two patients, we observed a significant elevation in WSS at, or proximal to, the region of the ICA’s sidewall where aneurysm formation eventually took place, suggesting potential anterograde formation in these patients. By using our computational pipeline, we may be able to differentiate between the pathophysiological pathways among patients, potentially influencing future treatment choices.

Once the structural changes in the vessel wall were initiated, CFD analysis revealed significantly elevated absolute WSSG near the proximal and distal boundaries of the bulging aneurysmal sac, which may further influence anterograde or retrograde development of the aneurysm. These findings may play a role in the transition from Type II to Type III blister aneurysms, which are characterized by berry-like and longitudinal sacs, respectively [5]. Furthermore, we hypothesize that the boundary of the longitudinal sac parallel to the parent artery represents the most significant area of high WSSG in Type III lesions, promoting circumferential growth into Type IV [5].

Fragile thin-walled and dissecting regions of unruptured intracranial aneurysms constitute areas susceptible to progression and rupture [24], which is an important consideration in the management of these lesions. Furthermore, it is known that areas of low WSS adjacent to regions characterized by high WSSG are associated with progression and bulging of the aneurysmal sac [25]. The CFD simulations presented support that initial blister aneurysm progression and dissection may be exacerbated by the combination of low intrasaccular WSS with high WSSG at the boundary of the aneurysm, where initial anterograde flow was observed (Fig. 6). This effect may subsequently contribute to further extension of the dissection in an anterograde or retrograde fashion after the initial stage of formation, notably influencing intrasaccular circulation patterns which can be anterograde flow or retrograde recirculation.

**Fig. 8:**
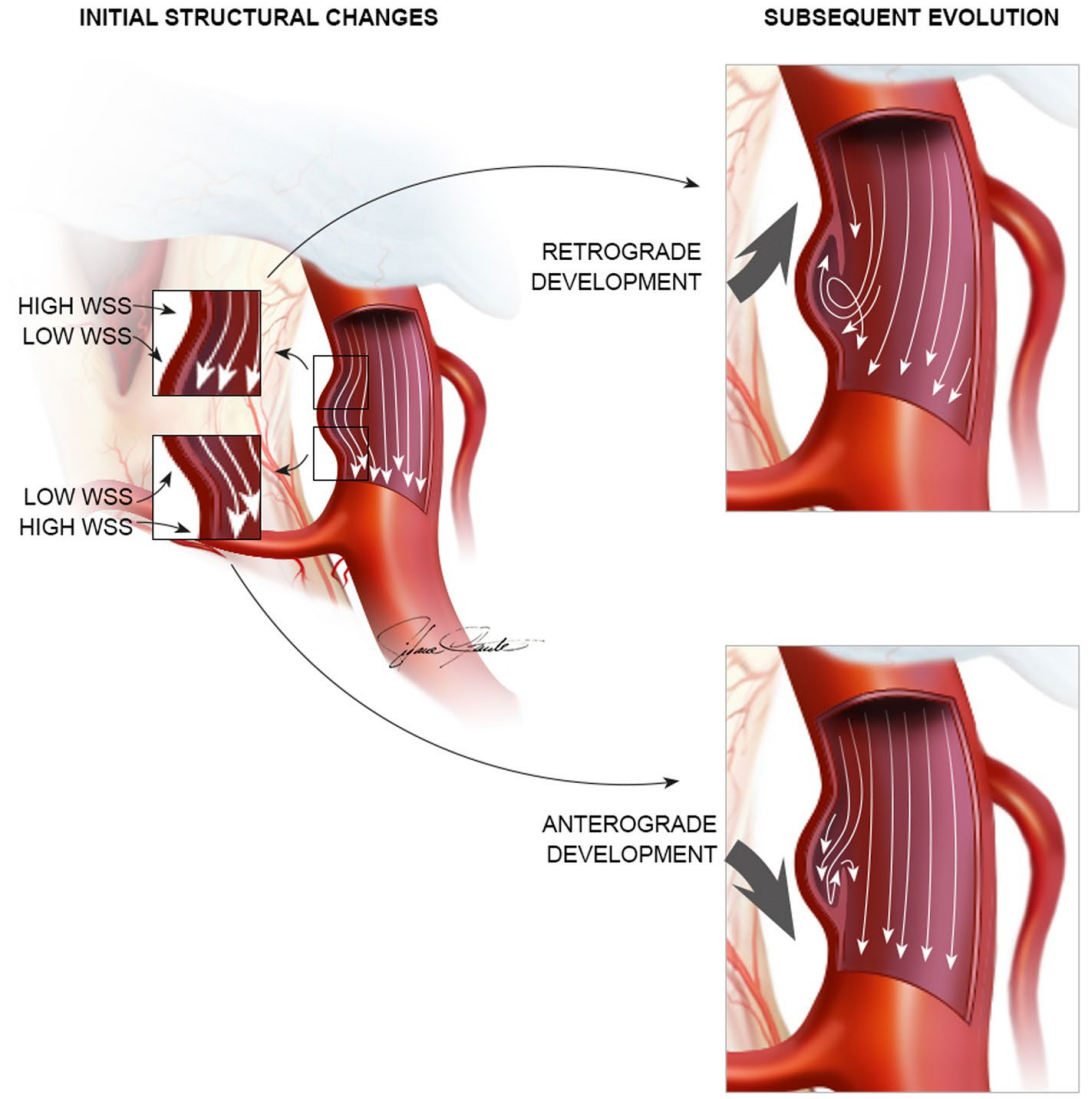
Illustration of initial structural changes at the proximal and distal neck of the aneurysm which may evolve in an anterograde or retrograde fashion. Very low intrasaccular WSS adjacent to high WSS in the parent artery wall may favor extension of the dissection after initial structures changes. The dissection further progresses either retrogradely with recirculating flow or anterogradely with anterograde flow in a larger aneurysmal pouch

We propose that the well-established intrasaccular retrograde recirculation flow patterns, as extensively documented in literature [25], are primarily induced by the initial aneurysmal morphology, which is heavily influenced by the early progression of the neck based on the WSSG. These flow patterns may play a significant role in extending the dissection of the proximal boundary of larger sidewall aneurysms, ultimately resulting in retrograde propagation of the dissection the following initial pathogenesis (Fig. 8). In instances of blister aneurysms forming in an anterograde fashion, our simulations suggest that the anterograde flow induces shearing forces along the distal boundary of the aneurysmal sac, thereby playing a role in its distal progression (Fig. 8). As the aneurysm grows, we hypothesize that the flow pattern aligns with the direction of dissection predicted by the correlation between the WSS distribution before aneurysm formation and the eventual location of the lesion. Although blister aneurysms are very rare and four cases provide valuable insights, additional cases are necessary to validate these mechanisms.

CFD techniques may potentially provide researchers with a means to identify the risk factors associated with intracranial aneurysms. By simulating blood flow patterns within the cerebral vasculature, CFD models can reveal hemodynamic parameters that contribute to intracranial aneurysm formation, progression, and potential rupture, which may allow for targeted interventions and personalized preventive measures.

CFD may also play a role in the development of novel treatments for intracranial aneurysms by providing detailed insights into the underlying flow dynamics and arterial mechanical stresses. Researchers may simulate the effects of different treatment modalities, such as stenting, coiling, or flow-diverting devices, to assess their efficacy and safety before clinical trials.

## Conclusions

Our simulations revealed that the geometry of the supraclinoid ICA may play a role in Type I and II blister aneurysm formation and progression by influencing the WSS distribution. In two out of four cases, WSS was significantly elevated on the dorsal wall of the supraclinoid segment of the ICA at the distal part of the future site of the aneurysm sac, suggesting that in some cases the aneurysm sac may ultimately develop in a retrograde fashion. In all cases, regions of supraphysiological WSS on the dorsal wall of the ICA was correlated with the future site of the blister aneurysm. Elevated WSSG at the proximal and distal boundaries of the bulging aneurysmal pouch may contribute to the transition between different blister morphologies. As the aneurysm grows, our study suggests that the blood flow pattern agrees with the direction of dissection predicted by the correlation between the WSS distribution before aneurysm formation and the eventual location of the lesion. These insights have implications for understanding the pathogenesis of these rare lesions and may help guide future treatment decisions.

## Methods

### Patient selection

Preoperative anteroposterior (AP) and lateral/oblique projections of X-ray angiographies or axial CT-angiographies were obtained from four patients with blister aneurysms located at non-branching sites along the dorsal part of the supraclinoid segment of the ICA. Additionally, the contralateral healthy ICA in a patient with a blister aneurysm, imaged by CT-angiography, served as an optimal control, eliminating the need for multivariate analysis since all background factors are identical between the healthy and pathological ICAs. All patients were treated with open surgery at our institution by the senior author (M.W.B).

### 3D modeling

Pertinent segments of the ICA were segmented using *lableme* [26] on all angiographic projections by the first author.

We developed a computer program to generate 3D reconstructions of the luminal iso-surfaces of the ICA using X-ray angiographies. This program was applied to three out of four patients with Type I and II blister aneurysms classified according to morphological classification [5]. A similar program was also developed for CT angiographic data for one patient.

**Fig. 9:**
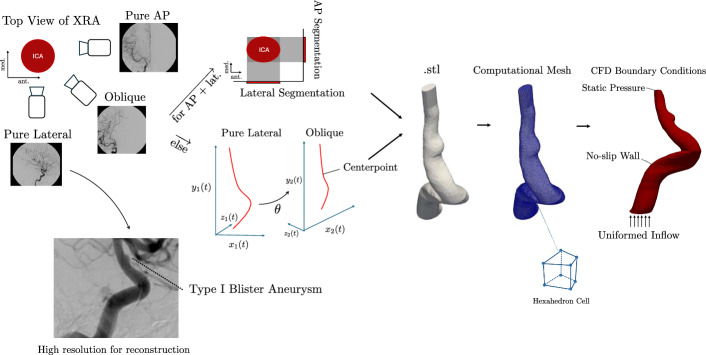
Illustration depicting the computational pipeline. Reconstruction algorithms for sets of high-quality perpendicular and oblique angiographic projections are outlined, enabling accurate and reliable reconstructions of blister aneurysms despite their small size. From this model, a mesh is generated using snappyHexMesh within the OpenFOAM framework for CFD meshing, resulting in a predominantly hexahedral mesh. The boundary conditions involving inlet flow and outlet pressure are illustrated

When pure lateral X-ray angiographic projections were available, we determined the luminal iso-surface of the vessel by fitting the largest ellipse at every axial slice of the ICA within the intersection of both orthogonal angiographic projections of the segmented vessel (Fig. 9).

When pure lateral projections were unavailable, we developed an algorithm to determine the angle $\uptheta$ separating AP and oblique projection planes. This was best approximated by computing the inverse cosine of the ratio of the ICA bifurcation angle on the oblique and AP views, which was evaluated on two angiographic cases of known angles to be accurate within 96%. This enabled us to reconstruct the ICA given input X-ray angiographies. Specifically, we let ${X}_{1}\left(t\right),{X}_{2}\left(t\right)$ be the 3D parametrized curves representing the center-point of every perpendicular cross-section of the healthy vessel along its course as seen in the AP and oblique views (Fig. 9). The center-point was computed with momentum averaging. While the 2D coordinates ${x}_{1}\left(t\right),{x}_{2}\left(t\right)$ and ${y}_{1}\left(t\right),{y}_{2}\left(t\right)$ were known from the X-ray angiographic projections, the depths ${z}_{1}\left(t\right),{z}_{2}\left(t\right)$ were computed as $argmi{n}_{{z}_{1}(t),{z}_{2}(t)}\left|\left|{X}_{2}\left(t\right)-{R}_{y}\left(\theta \right){X}_{1}\left(t\right)\right|\right|$. Subsequently, a circle of radius *R(t)* was added at all *t* values about the vessel spline to reconstruct the ICA in these cases, where *R(t)* was computed as the average of the radii measured on two angiographic projections. This approach is analogous to the method previously used in the 3D reconstruction of the coronary arteries from coronography [27]. Aneurysm reconstruction was added in Blender v3.4.1 (The Blender Foundation) using available angiographic projections such that the anterior and oblique projections of the model matched the angiographic data. Both methods for X-ray angiographic reconstruction of the ICA were evaluated by generating perpendicular and oblique projections of an open-source ICA model provided by SimVascular [28]. We then compared our software’s output against the ground truth. We also performed slicing of the 3D meshes along the Z-axis at 100 equidistant positions. Additionally, each mesh was centered and scaled to fit within a unit cube to standardize the analysis. For each slice, we computed the area of the 2D cross-section and estimated the uncertainty using a bootstrap resampling method with 300 iterations. This approach provided both the mean area and its associated standard deviation for each slice of every mesh. By analyzing the overlap between the uncertainty bands of the ground truth and reconstructed meshes, we assessed the consistency of the reconstructed geometries.

For CT-angiography, each axial slice of the ICA was segmented and reconstructed with the appropriate distance between axial CTA slices [29].

To model the boundary conditions, we added plates to the proximal inlet and distal outlets of the vessel with Blender. This software was also used for pre-mesh processing, notably to scale the model according to a standard ICA terminus [30]. Spikes and irregularities were manually removed, and holes were patched in Blender with care to respect luminal diameter, per Rayz et al.’s methodology [31]. To ensure that regions of interest near the outlet boundary were not artifactually influenced by its proximity, we extrapolated the supraclinoid ICA in patients 1 and 3.

Three-dimensional models of the ICA were also generated for each patient where the aneurysmal sacs were digitally removed from the parent artery using Blender surface smoothing and face reconstruction analogous to Gao et al.’s methodology [32] to simulate the hemodynamic conditions leading to the formation of these lesions. While it is known that aneurysm geometry undergoes changes around the time of rupture [33], our use of post-rupture patient imagery is inconsequential due to the digital removal of the lesion from the parent artery to probe the hemodynamic factors influencing blister aneurysm pathogenesis. In fact, contrary to the blister aneurysm itself, repeated conventional X-ray angiography following subarachnoid hemorrhage has revealed little to no change in ICA geometry with aneurysmal growth [14, 15, 34, 35].

Furthermore, our hemodynamic studies upon aneurysm formation are conducted to explore factors promoting dissection progression, a process which we analyze subsequent to blister aneurysm rupture. To evaluate our CFD methodology in addressing large sidewall aneurysms characterized by intrasaccular retrograde recirculation, we digitally increased the size of the aneurysmal sac in an illustrative blister aneurysm for comprehensive testing of larger sacs.

### CFD simulation

The pre-computational meshing and computational fluid dynamics were performed using OpenFoam 4.1 (OpenCFD Ltd) [36] with the Helyx OS v2.4.0 GUI (ENGYS Ltd) on a Linux machine running Ubuntu 14.04 (Canonical Ltd). We used snappyHexMesh within the OpenFOAM framework for CFD meshing, resulting in a predominantly hexahedral mesh. The basic mesh size was determined by ensuring that at least 25 evenly spaced cells spanned the artery's diameter, following the methodology previously established by Brambila-Solórzano et al. [37]. The mesh was refined at level (0 0), with maxLocalCells set to 500,000 and maxGlobalCells to 8,000,000. This produced meshes containing a minimum number of cells between 10^5^ to 10^6^, yielding a cell size of order 10^–4^ m to 10^–5^ m, which is a target in intracranial CFD studies [37].

Given the challenges of validating a CFD model for brain aneurysms, a mesh independence study was performed for all patients. We conducted a Grid Convergence Index (GCI) study using maximum WSS to confirm mesh independence in pre-aneurysmal models, as maximum WSS was the parameter of interest. Specifically, the GCI assesses the robustness of CFD solutions by quantifying how the results change with mesh refinement. To determine the convergence behavior, we computed the order of convergence *p* as:

$$p=\frac{\text{log}\left(\frac{{\upphi }_{3}-{\upphi }_{2}}{{\upphi }_{2}-{\upphi }_{1}}\right)}{\text{log}\left(r\right)},$$

where ${\phi }_{3}$, ${\phi }_{2},$ and ${\phi }_{1}$ are the maximum WSS values on successively finer meshes, respectively, and *r* is the refinement ratio between the meshes. To maintain GCI stability, we excluded the narrow range of WSS values near the inlet to account for non-physical proximal edge effects in both the control and patient no. 4.

The GCI is calculated using the following equation:

$${\text{GCI}}_{12}=\frac{1.25\times \left|\left({\upphi }_{2}-{\upphi }_{1}\right)/{\phi }_{2}\right|}{{\text{r}}^{\text{p}}-1}, {\text{GCI}}_{23}=\frac{1.25\times \left|\left({\phi }_{3}-{\phi }_{2}\right){/\phi }_{3}\right|}{{\text{r}}^{\text{p}}-1}.$$

This index quantifies the error due to mesh discretization and ensures that the numerical solution is independent of the mesh size, thereby verifying the reliability of the CFD results. We expect that ${\text{GCI}}_{23}={r}^{p}{\text{GCI}}_{12}$, where the asymptotic rate of convergence is assessed as ${\text{GCI}}_{23}/{r}^{p}{\text{GCI}}_{12}$, which should be close to 1.

While GCI < 5% is common [38, 39], we aimed for GCI < 3% for pathological ICAs. The mesh-independent cell sizes determined from the pre-aneurysmal cases were then applied to the aneurysmal CFD models for the same patients. The GCI values are summarized in Table 1, where the bold values indicate adequate GCI between the medium and fine meshing, showing sufficient grid independence. The finest mesh was used for the analysis.

**Table 1:** GCI mesh independence study for all patients

	Mesh level	Coarse	Medium	Fine
Control	# cells ($\times {10}^{5}$)	2.01	2.64	3.44
	Max WSS (Pa)	5.34	5.86	6.01
	GCI (%)	N/A	8.93%	**4.15%**
	Refinement ratio	1.31		
	Convergence order	2.67		
	Asymptotic score	1.04		
Patient no.1	# cells ($\times {10}^{5}$)	3.20	4.26	5.46
	Max WSS (Pa)	10.03	10.49	10.71
	GCI (%)	N/A	5.27%	**2.60%**
	Refinement ratio	1.31		
	Convergence order	2.60		
	Asymptotic score	1.02		
Patient no.2	# cells ($\times {10}^{5}$)	1.96	3.02	4.80
	Max WSS (Pa)	11.93	12.43	12.63
	GCI (%)	N/A	3.34%	**1.31%**
	Refinement ratio	1.57		
	Convergence order	2.06		
	Asymptotic score	1.02		
Patient no.3	# cells ($\times {10}^{5}$)	2.75	4.31	6.95
	Max WSS (Pa)	13.47	14.12	14.17
	GCI (%)	N/A	0.523%	**0.0432%**
	Refinement ratio	1.59		
	Convergence order	5.37		
	Asymptotic score	1.004		
Patient no.4	# cells ($\times {10}^{5}$)	1.36	1.94	2.70
	Max WSS (Pa)	36.19	47.07	48.69
	GCI (%)	N/A	5.05%	**0.73%**
	Refinement ratio	1.41		
	Convergence order	5.55		
	Asymptotic score	1.03		

Blood flow was modeled as an incompressible Newtonian fluid [40]. We set the dynamic viscosity to $\upmu$ = 0.0035 Pa·s and the blood density to 1056 $kg/{m}^{3}$ [41, 42]. Given Reynold’s number on the order of 500–1000, based on the input flow rate, density, viscosity, and ICA diameter between 3–5 mm for all patients, which suggested laminar flow, we used a steady-state laminar model. We employed the SimpleFoam solver with relaxation factors *p* = 0.3 and *U* = 0.7 to ensure model stability and convergence. Moreover, we used the Gauss linear scheme to discretize the gradient, divergence, and Laplacian terms, with the advected properties at cell faces computed using a bounded *linearUpwind* scheme for convective terms.

We assumed the arterial wall to be rigid, and a no-slip condition was enforced. Recent research indicates the rigid wall assumption yields results nearly identical to those obtained using fluid–structure interaction across various foundation stiffness coefficients in cases of atherosclerotic ICAs without stenosis [43], as is the case for the ICAs featured in our series.

Hemodynamic parameters rapidly and smoothly converged to a steady-state solution. Constant inlet flow rendered the simulations tractable for high spatiotemporal resolution without sacrificing model accuracy given that the pulsatile effects over a cardiac cycle have been shown to have a minor impact on time-averaged flow patterns [44–49]. Yet, a limitation of this methodology is that steady flow does not adequately account for flow disturbances present in the hemodynamic environment alongside the magnitude of WSS.

For our boundary value problem, we assumed at the inlet a zero-pressure gradient and a mean volumetric flow rate of 0.000004 ${m}^{3}/s$ [50]. We made used of the *flowRateInletVelocity* standard boundary condition in OpenFoam, which generates a uniform velocity profile perpendicular to the inlet. At the outlet on the other hand, we set the velocity gradient to zero and a mean arterial pressure profile of 90 mmHg. To ensure rapid convergence, pressure and velocity were initialized using potential flow.

## Data Availability

Anonymized data will be provided upon reasonable request to the corresponding author.
